# Patient-reported wellbeing and clinical disease measures over time captured by multivariate trajectories of disease activity in individuals with juvenile idiopathic arthritis in the UK: a multicentre prospective longitudinal study

**DOI:** 10.1016/S2665-9913(20)30269-1

**Published:** 2020-12-04

**Authors:** Stephanie J W Shoop-Worrall, Kimme L Hyrich, Lucy R Wedderburn, Wendy Thomson, Nophar Geifman, CAPS the CLUSTER Consortium, Eileen Baildam, Michael Barnes, Michael W Beresford, Emil Carlsson, Alice Chieng, Coziana Ciurtin, Gavin Cleary, Joyce Davidson, Fatjon Dekaj, Sally-Anne Dews, Andrew Dick, Gil Diogo, Teresa Duerr, Joanna Fairlie, Helen Foster, Jenna F Gritzfeld, Yiannis Ioannou, Beth Jebson, Melissa Kartawinata, Toby Kent, Aline Kimonyo, Saskia Lawson-Tovey, Wei-Yu Lin, Paul Martin, Flora McErlane, Fatema Merali, Andrew Morris, Helen Neale, Jessica Neisen, Sandra Ng, Elizabeth Ralph, Athimalaipet V Ramanan, Soumya Raychaudhuri, Emily Robinson, Samantha Smith, Emma Sumner, Damian Tarasek, Chris Wallace, Zoe Wanstall, Annie Yarwood

**Affiliations:** a Centre for Health Informatics, Manchester Academic Health Sciences Centre, University of Manchester, Manchester, UK; b Centre for Epidemiology Versus Arthritis, Manchester Academic Health Sciences Centre, University of Manchester, Manchester, UK; c Centre for Genetics and Genomics Versus Arthritis, Manchester Academic Health Sciences Centre, University of Manchester, Manchester, UK; d National Institute for Health Research Manchester Biomedical Research Centre, Manchester University Hospitals NHS Foundation Trust, Manchester, UK; e Centre for Adolescent Rheumatology Versus Arthritis at UCL, University College London Hospitals and Great Ormond Street Hospital, London, UK; f Infection Inflammation and Rheumatology, UCL Great Ormond Street Institute of Child Health, London, UK; g NIHR Great Ormond Street Hospital Biomedical Research Centre, London, UK

## Abstract

**Background:**

Juvenile idiopathic arthritis (JIA) is a heterogeneous disease, the signs and symptoms of which can be summarised with use of composite disease activity measures, including the clinical Juvenile Arthritis Disease Activity Score (cJADAS). However, clusters of children and young people might experience different global patterns in their signs and symptoms of disease, which might run in parallel or diverge over time. We aimed to identify such clusters in the 3 years after a diagnosis of JIA. The identification of these clusters would allow for a greater understanding of disease progression in JIA, including how physician-reported and patient-reported outcomes relate to each other over the JIA disease course.

**Methods:**

In this multicentre prospective longitudinal study, we included children and young people recruited before Jan 1, 2015, to the Childhood Arthritis Prospective Study (CAPS), a UK multicentre inception cohort. Participants without a cJADAS score were excluded. To assess groups of children and young people with similar disease patterns in active joint count, physician's global assessment, and patient or parental global evaluation, we used latent profile analysis at initial presentation to paediatric rheumatology and multivariate group-based trajectory models for the following 3 years. Optimal models were selected on the basis of a combination of model fit, clinical plausibility, and model parsimony.

**Finding:**

Between Jan 1, 2001, and Dec 31, 2014, 1423 children and young people with JIA were recruited to CAPS, 239 of whom were excluded, resulting in a final study population of 1184 children and young people. We identified five clusters at baseline and six trajectory groups using longitudinal follow-up data. Disease course was not well predicted from clusters at baseline; however, in both cross-sectional and longitudinal analyses, substantial proportions of children and young people had high patient or parent global scores despite low or improving joint counts and physician global scores. Participants in these groups were older, and a higher proportion of them had enthesitis-related JIA and lower socioeconomic status, compared with those in other groups.

**Interpretation:**

Almost one in four children and young people with JIA in our study reported persistent, high patient or parent global scores despite having low or improving active joint counts and physician's global scores. Distinct patient subgroups defined by disease manifestation or trajectories of progression could help to better personalise health-care services and treatment plans for individuals with JIA.

**Funding:**

Medical Research Council, Versus Arthritis, Great Ormond Street Hospital Children's Charity, Olivia's Vision, and National Institute for Health Research.

## Introduction

Juvenile idiopathic arthritis (JIA) is a heterogeneous condition with onset in childhood or early adolescence and common disease features that include joint swelling and pain.^1^ If not controlled, persistent joint inflammation can lead to cartilage damage and the potential need for joint-replacement surgery, with persistent pain and fatigue associated with functional limitations and lower health-related quality of life.^2^, ^3^

Key outcomes for individuals with JIA are included in a core outcome set and are incorporated into the juvenile arthritis disease activity score (JADAS) and clinical JADAS (cJADAS),^4^, ^5^ two composite outcome measures of JIA. Although JADAS allows for a single measure of disease activity in individuals with JIA, the individual components of the score do not always correlate.^6^, ^7^ This is particularly evident for components measured by the physician (active joint count or the physician's global assessment) versus those reported by the patient (such as parent or patient global evaluation). Approximately a quarter of children and young people (aged younger than 16 years) with clinically inactive disease (a disease state defined by clinician-only measured markers of disease activity) have ongoing symptoms,^8^ including disability, in the absence of synovitis.^9^ In the longer term, these persistent symptoms are associated with lower functional ability and health-related quality of life, even when inflammation is controlled at an early stage of the disease.^3^

**Research in context**

**Evidence before this study**

Traditionally, outcomes in chronic disease research, such as those used in juvenile idiopathic arthritis (JIA), have focused on changes either in single disease measures or in composite outcomes over time. This does not allow an understanding of how individual measures change in relation to each other. Because most measures represent unique aspects of disease, one cannot assume that they change in parallel. Novel unsupervised machine learning methods offer the opportunity to find so-called latent clusters of outcomes over time, facilitating a greater understanding of disease progression than is possible with a traditional dichotomy of improved or not improved. We searched MEDLINE and Embase from April 1, 1974, to Jan 1, 2019, for studies published in English of JIA (MeSH “juvenile arthritis”) using the search strings (MeSH “machine learning” or “artificial intelligence” or key word “trajectory”). Published studies have identified clusters of children and young people that differ in single aspects of disease over time. We did not find studies that used longitudinal multivariate approaches to understand how core clinical outcomes in patients with JIA progress in relation to each other after diagnosis.

**Added value of this study**

Our work reports global longitudinal patterns of disease in children and young people with JIA, extending knowledge of the heterogeneity in disease course beyond the existing International League of Associations for Rheumatology paradigm. Additionally, studies have repeatedly shown that patient-reported outcomes do not correlate well with physician-reported outcomes. Our study shows that there are multiple clusters among children and young people with JIA who have different patterns in these outcomes over time, and that these patterns sometimes diverge. In doing so, we provide a foundation for reassessment of how JIA disease measures are used to capture disease course and clinical outcomes.

**Implications of all the available evidence**

These data show that although disease severity at onset of JIA is important across several JIA core outcomes, it does not entirely predict future disease course, particularly for those who have persistently poor wellbeing despite improvement in clinically detected arthritis. These children and young people have a large, unmet need for early identification and personalised management plans, because disease in these individuals is often difficult to manage with anti-rheumatic therapies alone. Our study also shows that novel unsupervised machine learning methods applied to traditional epidemiological data represent an exciting step toward stratified management for children and young people with JIA, as new avenues for biomarker discovery and further understanding of disease mechanisms become available within this heterogeneous disease.

To further personalise medicine in JIA, it is important to understand the global patterns of disease, including how signs and symptoms of disease manifest at diagnosis, and how they unfold thereafter. Novel unsupervised machine learning methods are able to sort through heterogeneous real-world data and group individuals into previously unrecognised latent classes, in which shared disease patterns are identified among multiple signs and symptoms, either at a single time point (cross-sectionally) or longitudinally over time.^10^, ^11^ Instead of producing dichotomous, low-versus-high or improved-versus-not-improved groups, unsupervised machine learning algorithms can identify groups with unique profiles regarding rates or patterns of improvement,^12^ or groups that differ with regard to the relationships between multiple disease manifestations over time.^13^ Understanding these patterns of disease and the characteristics of subpopulations of children and young people belonging to each class might aid the forecasting of disease activity and personalised treatment of patients with JIA, by providing more accurate stratification of the population.

To identify potential distinct groups within children and young people with JIA, several methods have been applied. Traditionally, consensus-based techniques have been used to develop JIA classification criteria.^14^, ^15^, ^16^, ^17^ These are largely based on joint counts and extra-articular manifestations, in addition to a small number of biomarkers. Compared with these approaches, there have been fewer attempts to identify clusters of JIA over time based on core outcome variables or other measures. Using longitudinal clustering statistics, studies have identified five distinct groups on the basis of active joint count,^18^ three to five groups on the basis of pain,^19^, ^20^ and four to five groups on the basis of health-related quality of life^21^ over time. However, these studies focused on single aspects of disease, and thus they do not provide information on how these factors evolve over time in relation to each other.

The aim of this analysis was to use unsupervised machine learning approaches to identify unique clusters of children and young people with JIA, both at first presentation to paediatric rheumatology and during a 3-year follow-up. To our knowledge, ours is the first analysis to use cross-sectional and longitudinal multivariate approaches that include data across multiple core clinical outcomes.

## Methods

### Study population

The study population was comprised of children and young people recruited for the Childhood Arthritis Prospective Study (CAPS), a UK multicentre inception cohort of patients with childhood-onset arthritis. CAPS began recruitment in 2001 and had recruited over 1700 participants by Jan 1, 2019. Ethical approval for the cohort was gained from the Northwest Multicentre Ethics Committee (REC/02/8/104, IRAS 184042); written informed consent was provided by the parents or guardians, and assent was provided from children and young people, where appropriate.

Children and young people with a physician's diagnosis of JIA were selected for analysis if they were recruited before Jan 1, 2015, to allow for at least 3 years of follow-up. If no cJADAS score could be calculated at any point, they were excluded.

### Data collection

Data were collected at initial presentation to paediatric rheumatology (baseline) and annually thereafter. Between 2001 and 2010, data were also collected 6 months after baseline. Data at each timepoint included demographics, disease features including International League of Associations for Rheumatology (ILAR) category, disease activity, and medication data; these were extracted from medical records by study nurses. For this analysis, ILAR categories were assigned using data recorded 1 year after baseline; if this was missing, the closest ILAR category recorded on either side of 1 year was used, with preference given for the baseline value. Additionally, participants were asked to complete the Childhood Health Assessment Questionnaire (CHAQ), which incorporates two 100 mm visual analogue scales (VAS) for pain and patient or parent global evaluation of wellbeing. Parents or guardians of participants younger than 11 years completed these questionnaires; the option to self-complete was available for young people aged 11 years or older.

An index of multiple deprivation ranking was assigned to all children and young people on the basis of their residence (postcode) at the time of study registration; these rankings were based on the English indices of deprivation, the Welsh index of multiple deprivation, and the Scottish index of multiple deprivation.^22^, ^23^, ^24^ Within each country (England, Scotland, and Wales), deprivation is ranked on the basis of a combination of seven (eight in Wales) domains of deprivation (income, employment, health, education, crime and community safety, housing, living environment, and access to services) and then split into quintiles. Each child and young person was mapped to the relevant quintile within their country on the basis of their rank, and then the quintiles for all three countries were combined into a single variable (quintiles) for analysis.

### Modelled disease outcomes

The outcome measures used for the primary analysis were the components of the cJADAS with an active joint count up to ten (cJADAS 10): active joint count, physician's global assessment of disease activity, and patient or parent global evaluation of wellbeing. Components of cJADAS 10 were chosen in preference to JADAS because the cJADAS 10 does not require erythrocyte sedimentation rate, which is often not measured routinely in children with JIA in the UK.^5^, ^25^ VAS scores were converted from mm to cm, in a range of 0–10 cm. As part of cJADAS 10, an active joint count up to ten was used. This score was used because of high correlation with cJADAS 27 and 71,^5^ while reducing data skew and allowing all outcomes to fall on a common scale.

### Clustering and subgroup discovery

We used two clustering approaches to explore latent classes in patients with JIA: a cross-sectional analysis at baseline and a longitudinal analysis from baseline to 3 years of follow-up. Longitudinal clustering was undertaken independently of baseline clustering, with no inheritance of classes between the two methods. Class assignment at baseline versus in trajectories over 3 years were then compared graphically by use of a chord diagram, which illustrates the mapping between the two resulting sets of classes or clusters.

Latent profile analysis (a form of generalised structural equation modelling that fits categorical latent variables based on continuous observed variables) identified subgroups of children and young people on the basis of shared cJADAS 10 components at baseline. Latent profile analysis was applied with STATA 15 package gsem. Each individual was exclusively assigned to a cluster for which the highest posterior probability of group membership was obtained. Models used Poisson (for active joint count) and normal (for global scores) distributions. We selected the optimal number of groups (out of ten) on the basis of the Bayesian information criteria (BIC), selecting that which resulted in the lowest BIC.

Group-based trajectory models (specialised longitudinal finite mixture models within latent class growth analyses, with model-estimated parameters based on maximum likelihood estimation)^26^ were then used to group children and young people on the basis of shared trajectories of cJADAS 10 components during the 3 years after initial presentation to paediatric rheumatology. These models were constructed with STATA package traj. The model was specified with the three cJADAS 10 components as the dependant or outcome variables, using censored-normal distributions for both global assessments and zero-inflated Poisson distribution for active joint count. We tested linear, quadratic, and cubic polynomials for the trajectory shape, with one to ten trajectory groups tested within each polynomial form.^12^

We selected an optimal model through a combination of statistical fit, clinical plausibility, and parsimony. Initially, models were excluded if they had a group representing less than 1% of the cohort, a mean posterior probability for any group membership lower than 70%, or relative entropy lower than 0·5.^12^ The top models for each polynomial form were then selected on the basis of BIC. In cases where similar model fit and adequacy were evident between competing models, we selected between similarly fit models by assessing “clinical characterisation and plausibility”, as recommended by Lennon and colleagues.^12^

### Characteristics of clusters

We examined characteristics of children and young people such as demographic, clinical, and psychosocial factors at initial presentation for clusters within the latent classes at baseline and the trajectory groups using descriptive and univariable statistics. Additionally, pain (10 cm VAS) and functional ability (CHAQ scores) were compared with component trajectories in the optimal model, as these variables have previously been found to explain variation in wellbeing scores in both individuals with JIA^27^ and those with rheumatoid arthritis.^28^ We assessed differences in categorical variables with χ^2^ and Fisher's exact test and continuous variables with Kruskal-Wallis statistics for longitudinal models.

### Missing data

We undertook no imputation of missing data. As a maximum-likelihood-based technique, group-based trajectory modelling is somewhat robust to potential bias caused by missing data, as long as they are missing at random.^29^ This assumption of data missing at random was explored in additional analyses detailed in the appendix (pp 9–11). All analyses were done with Stata version 14, except for latent profile analyses, done with Stata version 15. Data visualisation was completed in R, version 3.6.1, using RStudio, version 1.2.5001.

### Sensitivity to noise

We did a secondary analysis to evaluate the sensitivity of the longitudinal trajectory model to random noise added to the dataset. Random noise was taken from a zero-inflated Poisson distribution (λ=2, p_0_=0·4) for active joint count and from a censored normal distribution with bounds at 0 and 10 by use of the STATA gentrun package for physician and parental global scores. Trajectories were evaluated after adding 1%, 2·5%, 5%, 10%, and 25% additional noise to the dataset.

### Role of the funding source

The funder of the study had no role in study design, data collection, data analysis, data interpretation, or preparation of this manuscript. The corresponding author had full access to all data in the study and had final responsibility for the decision to submit for publication.

## Results

Between Jan 1, 2001, and Dec 31, 2014, 1423 children and young people with JIA were recruited to CAPS. 238 participants without a record of cJADAS 10 were excluded, and one participant died during the study, therefore the final analysis included 1184 children and young people. Compared with those excluded, the study cohort was younger (by a median 1·7 years), had a higher proportion of white participants (1044 [90%] of 1166 *vs* 178 [79%] of 225), and a higher proportion of participants with rheumatoid factor-negative polyarthritis (265 [23%] of 1179 *vs* 27 [12%] of 222; appendix pp 1–2).

773 (65%) of 1184 participants were female and 411 (35%) were male, and 594 (50%) of 1179 with available ILAR category data had oligoarthritis (table 1). Median age at initial presentation was 7·4 years (IQR 3·4–11·7). Over the course of 3 years, 205 children and young people were discharged from paediatric rheumatology, with reasons including being “well” (80 [39%]), repeat non-attendance (26 [13%]), and transfer to adult service (59 [29%]; appendix p 1). Overall, cJADAS 10 data were available for 1140 (96%) of children and young people at baseline and for 912 (77%) at 6-month, 1116 (94%) at 1-year, 1030 (87%) at 2-year, and 949 (80%) at 3-year follow-ups.

**Table 1:** Baseline characteristics

			**Participants (n=1184)**	**Data available, n (%)**
Demographic features				
	Gender		..	1184 (100%)
		Female	773 (65%)	..
		Male	411 (35%)	..
	Race or ethnicity		..	1166 (98%)
		White	1044 (90%)	..
		Other race or ethnicity	122 (10%)	..
	Age at initial presentation, years		7·4 (3·4–11·7)	1174 (99%)
	Symptom duration to initial presentation, months		4·5 (2·2–8·6)	1156 (98%)
	Socioeconomic status (IMD)		..	1107 (93%)
		20% most deprived	295 (27%)	..
		Middle 60%	592 (53%)	..
		20% least deprived	220 (20%)	..
ILAR category			..	1177 (99%)*
	Systemic		71 (6%)	..
	Oligoarthritis			
		Persistent	528 (45%)	..
		Extended	66 (6%)	..
	RF-negative polyarticular		265 (23%)	..
	RF-positive polyarticular		45 (4%)	..
	Enthesitis-related		69 (6%)	..
	Psoriatic		85 (7%)	..
	Undifferentiated		50 (4%)	..
cJADAS 10 components				
	Active joint count		2 (1–5)	1092 (92%)
	Physician's global assessment, cm		2·9 (1·6–5·1)	855 (72%)
	Patient or parent global evaluation, cm		2·3 (0·6–5·0)	867 (73%)
	Overall cJADAS 10 score		9·0 (4·8–14·3)	672 (57%)
Other disease-related variables				
	Limited joint count		1 (1–4)	1092 (92%)
	ESR		21 (8–49)	831 (70%)
	CHAQ		0·8 (0·1–2·0)	868 (73%)
	Pain evaluation, cm		3·0 (0·9–5·8)	869 (73%)
	Uveitis (at presentation)		36 (4%)	858 (72%)
Treatment				
	Time to first definitive treatment, days†		18 (2–56)	1184 (100%)
Psychosocial factors				
	CHQ psychosocial score (subset n=468)		50 (39–56)	..
	GHQ score (subset n=613)		29 (22–38)	..

Data are n (%) or median (IQR), unless otherwise specified. CHAQ=Childhood Health Assessment Questionnaire. CHQ=Child Health Questionnaire. cJADAS 10=clinical Juvenile Arthritis Disease Activity Score with an active joint count up to ten. ESR=erythrocyte sedimentation rate. GHQ=General Health Questionnaire. ILAR=International League of Associations for Rheumatology. IMD=index of multiple deprivation. RF=rheumatoid factor.

* 84% taken from 1-year follow-up.

† Intra-articular steroids in oligoarthritis and methotrexate in other ILAR categories.

**Figure 1:**
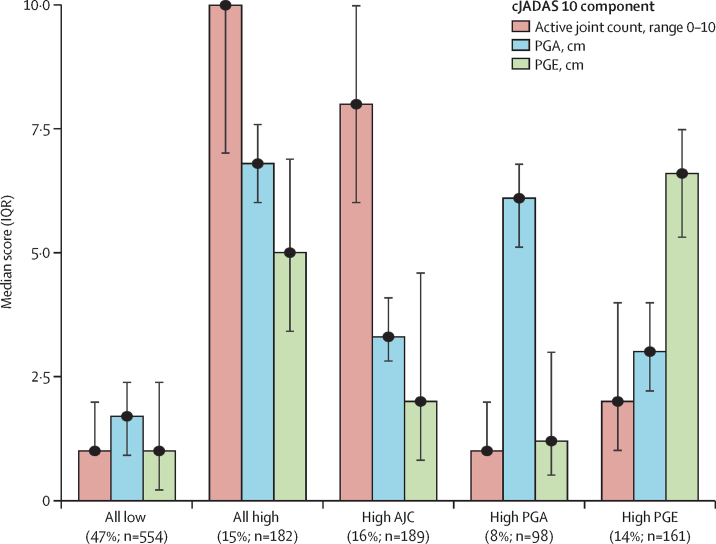
**Median cJADAS 10 components for each latent class at baseline** AJC=active joint count. cJADAS 10=clinical Juvenile Arthritis Disease Activity Score with an active joint count up to ten. PGA=physician's global assessment. PGE=patient or parent global evaluation.

The optimal model identified five distinct classes of children and young people at baseline (figure 1, average posterior probability for assigned group 0·78). The largest group (comprising 554 [47%] of 1184 participants) had lower values than those of other groups for all cJADAS 10 components (termed all-low group). A second group included 182 (15%) participants and had raised values in all three outcomes (termed all-high group). Three other groups were characterised by low to moderate disease activity, with higher values in a single cJADAS 10 component than in the other two components: high active joint count (189 [16%] participants), high physician global assessment (98 [8%]), and high patient or parent global evaluation (161 [14%]). Clinical and demographic characteristics of these groups are presented in the appendix (pp 2–4).

**Figure 2:**
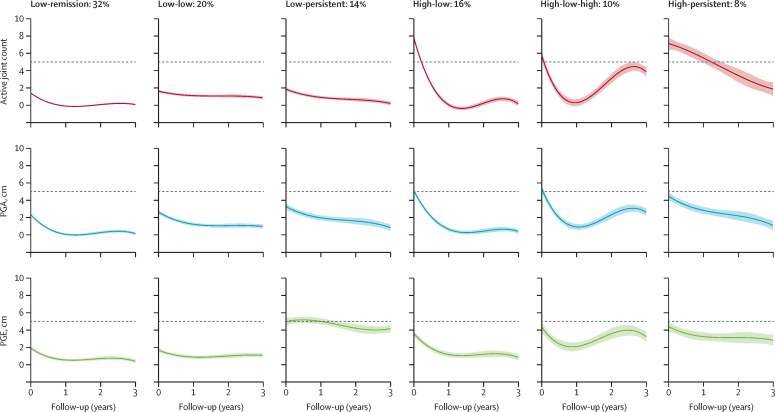
**Mean active joint count, PGA, and PGE trajectories within six multivariate disease clusters of children and young people with JIA over 3 years after an initial presentation to paediatric rheumatology** Error bands surrounding mean trajectories were constructed with use of the predict method for linear smoothed ggplots within R, version 3.6.1, stat_smooth function. JIA=juvenile idiopathic arthritis. PGA=physician's global assessment. PGE=patient or parent global evaluation.

The optimal multivariate model classified six cubic trajectory groups (figure 2; appendix pp 5–7). In four groups, the three outcomes followed approximately similar trajectory patterns and values, defined according to their relative patterns of severity across the outcomes from baseline to 3-year follow-up: low-remission group (380 [32%] of 1184 participants), low-low group (242 [20%]), high-low group (191 [16%]), and high-low-high group (113 [10%]). In the two remaining groups (low-persistent group and high-persistent group), the different components followed divergent trajectories. Children and young people in these groups either had lower initial joint counts and physician global scores that improved over time (low-persistent group, 162 [14%] participants) or higher initial joint counts and physician global scores that improved over time (high-persistent group, 96 [8%] participants). In both these groups, however, participants had an initially higher patient or parent global evaluation score that remained relatively static with little improvement over time (figure 2). When pain and function scores were visualised alongside the identified groups, these tended to follow similar patterns to wellbeing scores across the cohort (appendix p 8). All groups were stable up to an additional 2·5% noise. At 5% additional noise, the high-low-high group was less evident, and at 25% additional noise, the high-persistent group was replaced by a group with persistently high disease across all outcomes. Low-remission, low-low, low-persistent, and high-low groups were evident across all tested levels of additional noise (appendix pp 12–13).

We observed significant differences in sociodemographic variables between the six multi-trajectory groups, including higher age at onset and higher social deprivation score in participants in the low-persistent and high-persistent groups compared with those in the other groups (table 2).

**Table 2:** Demographic and clinical characteristics measured at initial presentation to paediatric rheumatology across multivariate trajectory groups

			**Low-remission**	**Low-low**	**High-low**	**High-low-high**	**Low-persistent**	**High-persistent**	p value*
Group population (n=1184)			380 (32%)	242 (20%)	191 (16%)	113 (10%)	162 (14%)	96 (8%)	..
Demographic features									
	Gender								
		Female	237 (62%)	164 (68%)	129 (68%)	81 (72%)	95 (59%)	67 (70%)	0·14
		Male	143 (38%)	78 (32%)	62 (32%)	32 (28%)	67 (41%)	29 (30%)	..
	Race or ethnicity								
		White	339 (91%)	211 (89%)	170 (90%)	96 (88%)	140 (87%)	88 (93%)	0·63
		Other race or ethnicity	35 (9%)	27 (11%)	19 (10%)	13 (12%)	21 (13%)	7 (7%)	..
		Patients with available data	374 (98%)	238 (98%)	189 (99%)	109 (96%)	161 (99%)	95 (99%)	..
	Age, years†		6·5 (2·9–11·0)	6·8 (3·1–11·0)	6·9 (3·4–11·0)	8·5 (4·5–12·0)	8·7 (3·7–13·0)	11·0 (7·2–14·0)	<0·0001
	Disease duration, months		4·2 (2·0–7·2)	4·0 (2·1–8·9)	3·8 (2·1–7·6)	5·9 (1·0–13·0)	5·2 (2·1–9·6)	7·0 (3·5–15·0)	<0·0001
Socioeconomic status			..	..	..	..	..	..	<0·0001
	20% most deprived		81 (22%)	50 (22%)	40 (24%)	32 (30%)	62 (42%)	30 (34%)	..
	Middle 60%		200 (55%)	121 (52%)	94 (55%)	55 (52%)	71 (48%)	50 (57%)	..
	20% least deprived		83 (23%)	60 (26%)	36 (21%)	19 (18%)	15 (10%)	7 (8%)	..
	Patients with available data		364 (96%)	231 (95%)	170 (89%)	106 (94%)	148 (91%)	88 (92%)	..
ILAR category									
	Systemic		21 (6%)	10 (4%)	19 (10%)	10 (9%)	7 (4%)	4 (4%)	<0·0001
	Oligoarthritis								
		Persistent	274 (72%)	145 (60%)	22 (12%)	14 (13%)	70 (44%)	3 (3%)	..
		Extended	8 (2%)	18 (7%)	10 (5%)	11 (10%)	14 (9%)	5 (5%)	..
	RF-negative polyarthritis		25 (7%)	32 (13%)	99 (52%)	46 (41%)	19 (12%)	44 (46%)	..
	RF-positive polyarthritis		3 (1%)	5 (2%)	10 (5%)	8 (7%)	7 (4%)	10 (10%)	..
	Enthesitis-related		15 (4%)	9 (4%)	8 (4%)	7 (6%)	18 (11%)	12 (13%)	..
	Psoriatic		20 (5%)	16 (7%)	10 (5%)	11 (10%)	19 (12%)	9 (9%)	..
	Undifferentiated		12 (3%)	6 (2%)	13 (7%)	5 (4%)	5 (3%)	9 (9%)	..
	Patients with available data		378 (99%)	241 (>99%)	191 (100%)	112 (99%)	159 (98%)	96 (100%)	..
Disease-related features									
	cJADAS 10		4·9 (3·0–8·2)	6·2 (3·7–8·3)	16·5 (13·1–19·8)	17·0 (12·5–19·9)	10·5 (7·7–12·7)	17·3 (12·8–20·5)	<0·0001
	Active joint count		1 (1–2)	1 (1–2)	9 (6–14)	5 (3–10)	1 (1–3)	9 (4–21)	<0·0001
	Limited joint count		1 (0–2)	1 (1–2)	5 (2–10)	4 (1–8)	1 (1–2)	6 (2–13)	<0·0001
	Physician's global assessment, cm		2·0 (1·0–3·1)	2·4 (1·2–3·8)	5·2 (3·2–7·0)	5·6 (3·7–7·0)	2·8 (1·8–4·7)	4·6 (2·8–6·4)	<0·0001
	Patient or parental global evaluation, cm		1·2 (0·2–3·0)	1·1 (0·3–2·6)	3·4 (1·1–6·0)	4·7 (1·8–6·3)	5·1 (3·1–7·0)	4·7 (2·2–6·5)	<0·0001
	CHAQ score		0·4 (0·0–0·9)	0·4 (0·0–0·9)	1·0 (0·5–1·8)	1·3 (0·8–2·0)	1·1 (0·5–1·6)	1·3 (0·8–1·9)	<0·0001
	ESR, mm/h		14 (6–35)	19 (7–40)	30 (14–60)	32 (13–60)	21 (8–41)	25 (8–79)	<0·0001
	Pain, cm		1·6 (0·2–4·6)	2·0 (0·5–4·5)	3·9 (1·0–6·0)	5·0 (2·2–7·0)	5·0 (3·5–6·6)	5·1 (3·1–7·1)	<0·0001
Specific joint activity (right or left)									
	Ankle		57 (16%)	56 (25%)	138 (76%)	55 (54%)	37 (26%)	57 (66%)	<0·0001
	Cervical spine		1 (<1%)	2 (1%)	15 (8%)	4 (4%)	0 (0%)	10 (12%)	<0·0001
	Hip		9 (3%)	3 (1%)	25 (14%)	8 (8%)	3 (2%)	16 (19%)	<0·0001
	Wrist		30 (8%)	19 (8%)	109 (60%)	38 (40%)	14 (10%)	43 (50%)	<0·0001
	Patients with available data		353 (93%)	227 (94%)	181 (95%)	101 (89%)	140 (86%)	86 (90%)	..
Extra-articular features									
	Systemic features in systemic JIA (n=71)		19 (90%; n=21)	9 (90%; n=10)	17 (89%; n=19)	9 (90%; n=10)	7 (100%; n=7)	4 (100%; n=4)	..
	Enthesitis in enthesitis-related JIA (n=56)		3 (27%; n=11)	2 (22%; n=9)	3 (38%; n=8)	1 (17%; n=6)	4 (33%; n=12)	5 (50%; n=10)	..
	Psoriasis in psoriatic JIA (n=73)		6 (32%; n=9)	3 (21%; n=14)	3 (30%; n=10)	3 (30%; n=10)	4 (33%; n=12)	5 (63%; n=8)	..
	Uveitis (n=854)		16 (6%; n=256)	6 (3%; n=181)	4 (3%; n=138)	2 (2%; n=87)	5 (4%; n=116)	3 (4%; n=76)	0·50
Psychosocial features									
	CHQ psychosocial score (n=468)		51·0 (43·8–56·8)	52·5 (45·0–57·8)	48·8 (37·3–54·0)	42·3 (30·8–54·1)	46·8 (32·7–54·6)	45·4 (38·5–51·1)	<0·0001
	GHQ score (n=613)		28 (20–34)	28 (22–36)	30 (24–41)	32 (24–41)	31 (24–44)	33 (22–44)	0·0020
Treatment within 3-year follow-up									
	Time to first definitive treatment, days‡		18 (7–49)	25 (7–82)	10 (0–35)	14 (0–57)	28 (7–103)	6 (0–59)	<0·0001
	Ever biological use between initial presentation and 3-year follow-up		26 (7%)	47 (19%)	51 (27%)	53 (47%)	51 (31%)	54 (56%)	<0·0001

Data are n (%) or median (IQR). Percentages are out of available data for each variable (table 1). CHAQ=Childhood Health Assessment Questionnaire. CHQ=Child Health Questionnaire. cJADAS 10=clinical Juvenile Arthritis Disease Activity Score with a ten active joint count. ESR=erythrocyte sedimentation rate. GHQ=General Health Questionnaire. ILAR=International League of Associations for Rheumatology. JIA=juvenile idiopathic arthritis. RF=rheumatoid factor.

* Kruskal-Wallis, χ^2^, or Fisher's exact test.

† Age at initial presentation to paediatric rheumatology.

‡ Intra-articular steroids in oligoarthritis, synthetic disease-modifying anti-rheumatic drugs in all other categories.

**Figure 3:**
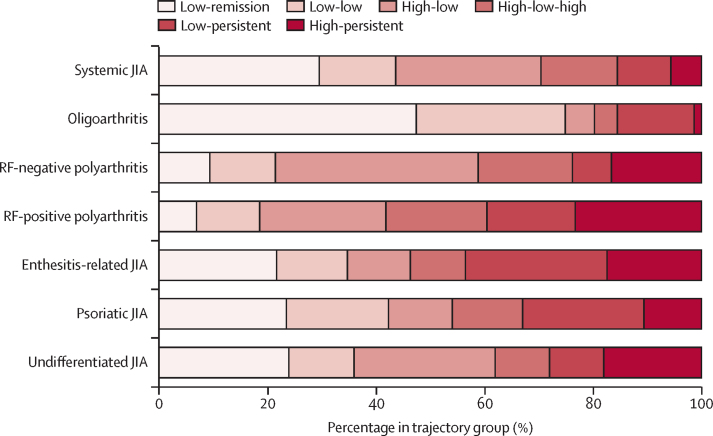
**Distribution of ILAR categories across the six cJADAS 10 multivariate trajectory groups** ILAR=International League of Associations for Rheumatology. cJADAS 10= clinical Juvenile Arthritis Disease Activity Score with an active joint count up to ten. JIA=juvenile idiopathic arthritis. RF=rheumatoid factor.

Each multivariate trajectory group included children and young people from all ILAR categories (figure 3). The majority of participants with oligoarthritis were assigned to the low-remission (282 [47%] of 594 participants) or low-low (163 [27%]) groups. Those with oligoarthritis assigned to the high-low group (32 [5%] of 594 participants) had median six active joints at presentation. Across ILAR categories, the highest proportions of children and young people assigned to the low-persistent trajectory were within enthesitis-related arthritis (18 [26%] of 69 participants) and psoriatic arthritis (19 [22%] of 85), whereas those with rheumatoid factor-positive polyarthritis had the highest assignment into the high-persistent trajectory group (12 [27%] of 45; figure 3). Additionally, those with persistent disease symptoms (low-persistent and high-persistent groups) had higher wellbeing scores at baseline (median patient or parental global evaluation score of 5·1 cm for low-persistent, median 4·7 cm for high-persistent) than those in other trajectory groups whose wellbeing scores remained low or improved over time (figure 3), alongside poorer health-related quality of life for both participants and parents.

**Figure 4:**
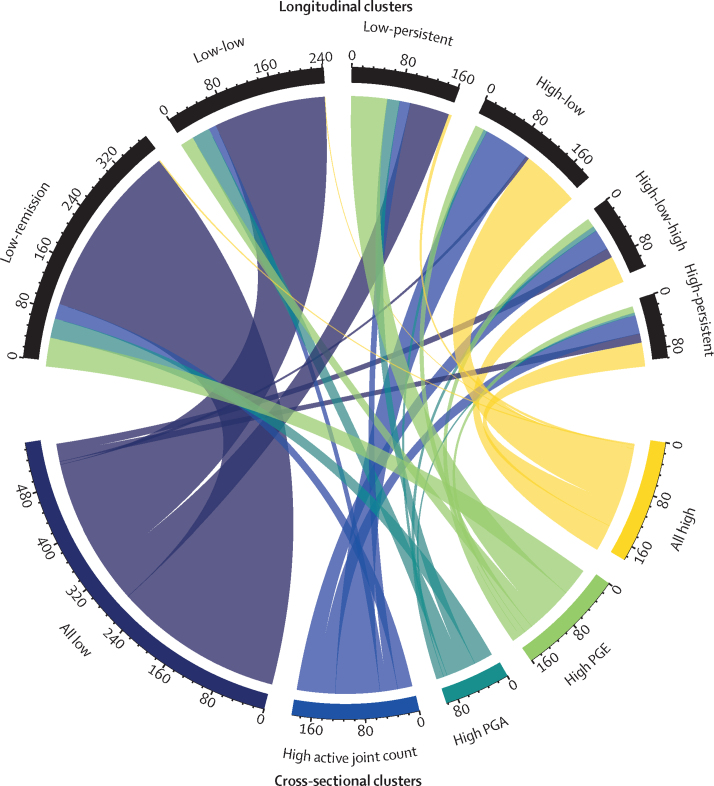
**Mapping from data-driven cross-sectional clusters at baseline (lower) to longitudinal clusters over the 3-year follow-up (upper)** PGA=physician's global assessment. PGE=patient or parent global evaluation.

Children and young people from the all-low baseline cluster (554 participants) were frequently classed within the low-remission (278 [50%]), low-low (179 [32%]), and low-persistent (63 [11%]) trajectory groups. Those from the all-high (182 participants) and high active joint count (189 participants) baseline clusters tended to be classed within the high-low (93 [51%] for all-high, 73 [39%] for high active joint count), high-low-high (43 [24%] for all-high, 33 [17%] for high active joint count) and high-persistent (38 [21%] for all-high, 30 [16%] for high active joint count) groups. The largest proportions of participants in the high patient or parent global group at baseline (161 participants) were classed within the low-persistent (57 [35%]) and low-remission (45 [28%]) trajectory groups (figure 4).

## Discussion

The signs and symptoms of JIA are diverse and can change over the course of disease, due to treatment effects and evolution of the underlying condition. Our study identified six clusters of children and young people with JIA who had different patterns across three core outcome variables in the 3 years after initial presentation to paediatric rheumatology. In four of these groups, the three components of cJADAS followed similar patterns to each other. In the two other groups, slow improvements in active joint counts and physician's global scores were coupled with persistent poor patient or parent wellbeing scores. When using information from baseline alone, five clusters of children and young people with JIA were identified; these overlapped to some degree, but not completely, with the six trajectory-based subgroups identified over follow-up. Therefore, disease severity at onset is important but is not entirely predictive of future disease state, which will also be influenced by interventions.

The classification criteria for JIA have been consensus-based and have largely focused on joint counts and extra-articular features in the early phase of disease.^14^, ^15^, ^16^, ^17^ ILAR category remains one of the strongest predictors of later remission.^30^, ^31^ In this study, we did not attempt to reclassify JIA; rather, using a data-driven approach, we tried to understand features of early disease. We report five clusters of JIA at baseline and six in the following 3 years, based on physician and patient assessment of disease. Although several ILAR categories had greater representation within certain disease clusters (eg, 64% oligoarthritis in the all-low baseline cluster, and 47% in the low-remission trajectory group), ILAR categories were not able to fully identify the outcomes of JIA over time. Similarly, clusters identified at baseline were not able to identify whether the three JIA outcomes included here would diverge from one another or remain in parallel over time. Longitudinal data-driven clustering was able to robustly capture baseline cluster patterns, while additionally capturing nuances of changing patterns across multiple disease measures over time. These longitudinal approaches are a promising avenue for stratified management plans in people with JIA, as they provide a more granular picture of disease activity and impact over time.

Our study reports six unique clusters of disease outcome over time. Multiple clusters of active joint counts were reported in a multicentre, retrospective cohort of 659 young people with JIA in Canada.^18^ This study reported high numbers of children and young people with low-remission (20% of participants) or low-low (45%) active counts, which is almost identical to the 66% of individuals in our study with minimal numbers of active joints over time. In our study, these low disease and remission groups were characterised by a high proportion of participants with oligoarthritis and a younger age than those in other groups. However, 14% of our cohort had persistent poor patient or parent global wellbeing scores despite low active joint counts (low-persistent group), constituting a distinguishing disease cluster and highlighting the usefulness of modelling multiple outcomes within a heterogonous disease. Focusing on disease symptoms, previous trajectory analyses have identified up to five groups of young people on the basis of health-related quality of life^21^ or pain.^19^, ^20^ Similar to the studies of Rashid and colleagues^19^ and Shiff and colleagues,^20^ both of which modelled pain trajectories, our study identified groups of children and young people with minimal symptoms, rapidly decreasing symptoms, or persistently high symptoms over time.^19^, ^20^ Of note, none of the groups identified in our study reached an average score of 0 cm for wellbeing, despite having an average of zero active joint counts for at least one timepoint in three of six groups (58% of the study population), suggesting that even within these low-activity groups, at least some children and young people did not rate their wellbeing score as zero.

To our knowledge, this was the first study to examine how the cJADAS 10 components track in relation to each other over time. The modelling of multiple measures of disease allowed for the identification of groups in which these scores diverged over time, which might have been less obvious if only the composite score had been used. Groups that had persistent poor wellbeing scores despite improving joint counts and physician global scores were difficult to distinguish from groups in which outcomes progressed in parallel to their initial presentation to paediatric rheumatology. Disease features at initial presentation were similar between groups that had low numbers of active joint counts at baseline and those that had high numbers of active joints, regardless of wellbeing trajectories. The greater representation of participants with enthesitis-related JIA in both persistent groups was a distinguishing disease feature (11% and 13% in persistent groups *vs* 4–6% in the other groups). Extra-articular features, such as enthesitis, are not captured in active joint counts, and these children and young people might have high related levels of inflammatory pain. Additionally, there might be substantial overlap between painful non-inflammatory conditions, such as fibromyalgia, and enthesitis,^32^, ^33^ which might not be used to inform the physician global assessment. Although similar clinically to participants in groups with improving wellbeing scores, those with persistently poor wellbeing scores tended to be older, have longer disease duration, live in more deprived areas, and have poorer health-related quality of life scores, with wellbeing scores at initial presentation higher in the low-persistent group (5·1 cm) than in the low-remission (1·2 cm) or low-low (1·1 cm) groups. High wellbeing scores at initial presentation coupled with low active joint counts might, therefore, indicate an individual already beginning on this divergent trajectory or with pre-existing health concerns. Using a different subset of patient-prioritised outcomes, Guzman and colleagues^34^ also reported a group where joint activity decreased despite persistent pain and impact on quality of life, including several study-defined impact measures. In the Canadian cohort,^34^ fewer children and young people were allocated to this disease course (10%) and they had relatively early control of joint activity, compared with the slow improvements observed in our study. Both of these groups of children and young people illustrate the heterogeneity in the signs and symptoms of JIA both over time and in relation to each other. This heterogeneity highlights the future applicability of unsupervised machine learning to inform stratified management approaches in children and young people with JIA, where groups with great unmet needs, such as those with persistent symptoms despite resolution of inflammatory joint activity, can be identified. A single composite score, such as cJADAS 10 score, might be an useful indicator of non-remission, but might not be able to, on its own, explain why a child or young person has not reached remission.

Our study benefitted from being set within one of the largest inception cohorts of children and young people with JIA globally. CAPS collects a wide range of information from both medical records and patient-completed questionnaires over time. This allowed for a detailed, longitudinal analysis of the cJADAS 10 components and the additional exploration of multiple factors, measured at initial presentation to paediatric rheumatology, that characterised group membership, such as demographic, clinical, and psychosocial characteristics. The inception nature of the cohort, together with models that could incorporate missing data, allowed for the inclusion of most of the cohort, minimising selection bias through both left-censorship and drop-out. Additionally, informative drop-out in CAPS allowed for the exploration of bias through data potentially missing not-at-random.

Our study assessed trajectories of disease from the point of initial presentation to paediatric rheumatology. All children and young people were treated within the same health-care system, but individual responses to treatment were not modelled. Some therapies might have been prescribed before initial presentation to paediatric rheumatology. Therefore, these trajectories do not necessarily reflect patterns from disease onset and are useful to understand progression from the point of first contact with paediatric rheumatology. The trajectories presented are means based on an approximately annual follow-up within the first 3 years after initial presentation to paediatric rheumatology. The JIA disease course might have increased variability over time with more frequent capture of disease measures. Additionally, modelling a higher active joint, such as in cJADAS 71, rather than cJADAS 10 would not be expected to produce additional trajectory groups but might have raised the initial mean joint counts for high-low, high-low-high, and high-persistent groups. Finally, the follow-up was limited to 3 years in this study, on the basis of the observation of greatest changes in disease over the first year after diagnosis,^3^ to limit bias from patients transferred to adult clinics and to maximise sample size. However, Berard^18^ highlighted the potential for disease worsening after 5–10 years of stable disease. Therefore, additional modelling would be valuable into adulthood, with effort needed in the research community to plan for the retention of individuals who have transferred to adult clinics.

Using the components of cJADAS, six distinct trajectories were observed for children and young people with JIA in the 3 years after initial presentation to paediatric rheumatology, with low disease activity and remission being common but not universal. Disease trajectories were not predicted entirely by ILAR category or disease manifestations at diagnosis. Importantly, in a fifth of children and young people, a divergence was observed between improving joint counts but persistently high scores for wellbeing, whose pain and function scores mirrored those of wellbeing over time. Understanding the biological and sociological mechanisms underpinning groups of children and young people within different clusters has the potential to improve disease management plans for a more personalised approach to treatment for individuals with JIA.

## Data sharing

Information regarding applying for access to CAPS data can be found online.
