# Correction to *Lancet Rheumatol* 2021; 3: e111–21

**DOI:** 10.1016/S2665-9913(21)00082-5

**Published:** 2021-03-24

*Shoop-Worrall SJW, Hyrich KL, Wedderburn LR, et al. Patient-reported wellbeing and clinical disease measures over time captured by multivariate trajectories of disease activity in individuals with juvenile idiopathic arthritis in the UK: a multicentre prospective longitudinal study.* Lancet Rheumatol *2021; **3:** e111–21*— In this Article, the name of Collaborator Gil Reynolds Diogo was incorrect. This correction has been made as of March 24, 2021.

