# Supplementary material for: Patient-reported wellbeing and clinical disease measures over time captured by multivariate trajectories of disease activity in individuals with juvenile idiopathic arthritis in the UK: a multicentre prospective longitudinal study
Source: Lancet Rheumatol. 2020 Dec 4;3(2):e111–21. doi: 10.1016/S2665-9913(20)30269-1 (PMC7843954; doi:10.1016/S2665-9913(20)30269-1)
Supplement: Supplementary appendix [file mmc1.pdf]

THE LANCET
Rheumatology

Supplementary appendix

This appendix formed part of the original submission and has been peer reviewed. We post it as supplied by the authors.

Supplement to: Shoop-Worrall SJW, Hyrich KL, Wedderburn LR, Thomson W, Geifman N, on behalf of CAPS and the CLUSTER Consortium. Patient-reported wellbeing and clinical disease measures over time captured by multivariate trajectories of disease activity in individuals with juvenile idiopathic arthritis in the UK: a multicentre prospective longitudinal study. *Lancet Rheumatol* 2020; published online Dec 4. https://doi.org/10.1016/S2665-9913(20)30269-1.

SUPPLEMENTARY MATERIALS

Supplementary Table 1. Characteristics of patients included in, and excluded from, the study

**Baseline variable**	**N (%) or median (IQR) of baseline variable**		**Univariable P-value (chi2/Mann-Whitney)**
	**Included**	**Excluded**	
N	1184	239	
Discharge reasons within the first three years (overall n=245)			0.091
Adult services	59 (29)	11 (18)	
Moved clinic	31 (15)	7 (18)	
Moved house	1 (<1)	0 (0)	
Well	80 (39)	9 (23)	
Repeat DNA	26 (13)	12 (30)	
Other	8 (4)	1 (3)	
Time to discharge (years)	2.2 (1.4, 3.4)	2.5 (1.5, 5.4)	0.1176
Age at initial presentation (years)	7.4 (3.4, 11.7)	9.1 (4.1, 12.6)	0.0172
Symptom duration to initial presentation (months)	4.5 (2.2, 8.6)	5.2 (2.5, 11.6)	0.0563
Female	773 (65)	146 (62)	0.264
White ethnicity	1044 (90)	178 (79)	<0.001
ILAR category			<0.001
Systemic	71 (6)	23 (10)	
Oligo (pers)	528 (45)	107 (48)	
Oligo (ext)	66 (6)	7 (3)	
RF- Poly	265 (23)	27 (12)	
RF+ Poly	43 (4)	7 (3)	
ERA	69 (6)	11 (5)	
PsA	85 (7)	12 (5)	

Undifferentiated	50 (4)	28 (13)	
Active joint count	2 (1, 5)	2 (1, 5)	0.3365
Physician global (cm)	2.9 (1.6, 5.1)	2.5 (1.1, 4.7)	0.0681
Parent global (cm)	2.3 (0.6, 5.0)	2.7 (0.4, 5.0)	0.9884

Supplementary Table 2. Model fit and percent classified into each group in baseline latent class analyses

Classes in model	BIC	Population probability of class membership (95% CI)									
		1	2	3	4	5	6	7	8	9	10
1	14176	1	-	-	-	-	-	-	-	-	-
2	12675	0.69 (0.66, 0.72)	0.31 (0.28, 0.34)	-	-	-	-	-	-	-	-
3	12658	0.56 (0.51, 0.60)	0.29 (0.26, 0.32)	0.15 (0.11, 0.20)	-	-	-	-	-	-	-
4	12634	0.48 (0.44, 0.53)	0.28 (0.25, 0.31)	0.16 (0.12, 0.20)	0.08 (0.05, 0.11)	-	-	-	-	-	-
5	12540	0.44 (0.39, 0.48)	0.16 (0.12, 0.20)	0.16 (0.13, 0.19)	0.14 (0.11, 0.17)	0.11 (0.09, 0.14)	-	-	-	-	-
6	12553	0.43 (0.39, 0.47)	0.15 (0.11, 0.20)	0.15 (0.12, 0.18)	0.14 (0.11, 0.18)	0.09 (0.07, 0.13)	0.03 (0.01, 0.07)	-	-	-	-
7*									-	-	-
8*										-	-
9*											-
10*											

*Models did not reach convergence after 10,000 iterations

Supplementary Table 3. Characteristics of CYP assigned to each baseline cluster

Characteristic	N (%) or Median (IQR) for each class				
	All Low	All High	High AJC	High PGA	High PGE

N (%)	554 (47)	182 (15)	189 (16)	98 (8)	161 (14)
**Outcome**					
Active joint count (0-10)	1 (1, 2)	10 (7, 10)	8 (6. 10)	1 (1, 2)	2 (1, 4)
Physician global (cm)	1.7 (0.9, 2.4)	6.8 (6.0, 7.6)	3.3 (2.8, 4.1)	6.1 (5.1, 6.8)	3.0 (2.2, 4.0)
Parent global (cm)	1.0 (0.2, 2.4)	5.0 (3.4, 6.9)	2.0 (0.8, 4.6)	1.2 (0.5, 3.0)	6.6 (5.3, 7.5)
**Demographics**					
Female	364 (66)	123 (68)	130 (70)	57 (58)	99 (61)
White or Caucasian	491 (90)	161 (90)	158 (87)	91 (94)	143 (90)
Age at initial presentation (years)	7.0 (3.3, 11.4)	8.4 (3.7, 12.1)	8.6 (4.0, 12.6)	5.9 (2.9, 11.4)	7.5 (3.3, 11.7)
Symptom duration to initial presentation (months)	4.6 (2.2, 8.5)	4.4 (2.3, 7.6)	5.4 (2.6, 11.4)	3.4 (1.6, 6.7)	3.8 (2.1, 10.0)
**Socioeconomic status (IMD)**					
20% most deprived	177 (25)	16 (28)	56 (26)	11 (32)	35 (40)
Middle 60%	387 (54)	34 (60)	116 (54)	13 (38)	43 (49)
20% least deprived	152 (21)	7 (12)	116 (54)	10 (29)	9 (10)
**ILAR Category**					
Systemic	20 (4)	20 (11)	13 (7)	14 (14)	4 (3)
Oligoarticular					
Persistent	354 (64)	9 (5)	24 (13)	58 (59)	83 (52)
Extended	26 (5)	16 (9)	24 (13)	8 (8)	8 (5)
RF- Polyarticular	59 (11)	98 (54)	80 (44)	5 (5)	23 (14)
RF+ Polyarticular	11 (2)	13 (7)	11 (6)	2 (2)	6 (4)

Enthesitis-related	27 (5)	5 (3)	15 (8)	6 (6)	16 (10)
Psoriatic	40 (7)	8 (4)	18 (10)	4 (4)	15 (9)
Undifferentiated	17 (3)	13 (7)	14 (8)	1 (1)	5 (3)
**Other disease-related variables**					
Limited joint count	1 (0, 2)	7 (3, 14)	5 (1, 9)	1 (1, 2)	2 (1, 3)
ESR	15 (6, 35)	40 (19, 77)	24 (10, 54)	20 (7, 60)	18 (8, 48)
CHAQ	0.4 (0.0, 0.9)	1.4 (0.9, 1.9)	0.8 (0.3, 1.5)	0.5 (0.1, 1.0)	1.3 (0.6, 1.6)
Pain	2.5 (0.7, 5.2)	5.7 (3.5, 8.0)	2.6 (0.5, 5.3)	4.0 (1.4, 6.6)	5.0 (2.9, 6.7)
**Treatment**					
Time to first definitive treatment (days)*	18 (4, 55)	20 (0, 57)	12 (0, 40)	25 (7, 78)	19 (0, 57)
**Psychosocial**					
CHQ psychosocial score (subset n=468)	51 (42, 57)	43 (32, 49)	50 (39, 57)	46 (31, 52)	38 (27, 50)
GHQ score (subset n=613)	29 (21, 36)	32 (22, 44)	28 (20, 36)	36 (23, 46)	37 (28, 52)

*AJC: Active joint count, PGA: Physician global, PGE: Parent global, ESR: Erythrocyte sedimentatino rate, RF: Rheumatoid Factor, CHAQ: Child Health Assessment Questionnaire, CHQ: Child Health Questionnaire, GHQ: General Health Questionnaire

Supplementary Table4. Model diagnostics across potential multi-trajectory models

Class numbers	AIC	BIC	Class proportions										Average posterior probability per class										Model average posterior probability for assigned class	Relative Entropy
			1	2	3	4	5	6	7	8	9	10	1	2	3	4	5	6	7	8	9	10		
**Linear**																								
**1**	-25539	-25569	1	0	0	0	0	0	0	0	0	0	1.00	-	-	-	-	-	-	-	-	-	1.00	1
**2**	-23832	-23887	0.67	0.33	0	0	0	0	0	0	0	0	0.96	0.94	-	-	-	-	-	-	-	-	0.95	0.90
**3**	-23395	-23477	0.34	0.46	0.20	0.00	0.00	0.00	0.00	0.00	0.00	0.00	0.85	0.91	0.94	-	-	-	-	-	-	-	0.90	0.76
**4**	-23297	-23405	0.01	0.59	0.18	0.21	0.00	0.00	0.00	0.00	0.00	0.00	0.81	0.93	0.90	0.92	-	-	-	-	-	-	0.89	0.80
**5**	-22794	-22927	0.15	0.36	0.20	0.18	0.11	0.00	0.00	0.00	0.00	0.00	0.83	0.87	0.84	0.90	0.93	-	-	-	-	-	0.87	0.67
**6**	-22785	-22944	0.01	0.14	0.36	0.11	0.18	0.20	0.00	0.00	0.00	0.00	0.74	0.83	0.87	0.93	0.89	0.83	-	-	-	-	0.85	0.66
**7**	-22575	-22760	0.07	0.07	0.30	0.23	0.15	0.06	0.13	0.00	0.00	0.00	0.86	0.86	0.83	0.80	0.89	0.91	0.83	-	-	-	0.85	0.66
**8**	-22564	-22709	0.01	0.08	0.23	0.07	0.28	0.14	0.13	0.05	0.00	0.00	0.68	0.84	0.80	0.85	0.82	0.88	0.83	0.92	-	-	0.83	0.57
**9**	-22490	-22652	0.01	0.05	0.06	0.28	0.23	0.08	0.11	0.12	0.05	0.00	0.68	0.79	0.87	0.83	0.81	0.79	0.86	0.82	0.90	-	0.82	0.55
**10**	-22536	-22674	0.01	0.00	0.05	0.28	0.06	0.23	0.08	0.11	0.05	0.12	0.68	0.55	0.80	0.82	0.87	0.81	0.79	0.86	0.90	0.82	0.79	0.54
**Quadratic**																								
**1**	-25217	-25258	1.00	0.00	0.00	0.00	0.00	0.00	0.00	0.00	0.00	0.00	1.00	-	-	-	-	-	-	-	-	-	1.00	1
**2**	-23542	-23620	0.68	0.32	0.00	0.00	0.00	0.00	0.00	0.00	0.00	0.00	0.97	0.93	-	-	-	-	-	-	-	-	0.95	0.89
**3**	-23181	-23297	0.33	0.46	0.21	0.00	0.00	0.00	0.00	0.00	0.00	0.00	0.85	0.91	0.93	-	-	-	-	-	-	-	0.90	0.75
**4**	-22720	-22873	0.40	0.29	0.20	0.11	0.00	0.00	0.00	0.00	0.00	0.00	0.89	0.86	0.89	0.95	-	-	-	-	-	-	0.90	0.72
**5**	-22729	-22918	0.00	0.29	0.40	0.11	0.20	0.00	0.00	0.00	0.00	0.00	0.79	0.86	0.89	0.95	0.89	-	-	-	-	-	0.87	0.69
**6**	-22345	-22571	0.16	0.14	0.13	0.39	0.07	0.11	0.00	0.00	0.00	0.00	0.83	0.84	0.87	0.88	0.94	0.85	-	-	-	-	0.87	0.63

**7**	-22347	-22610	0.11	0.26	0.07	0.39	0.10	0.06	0.00	0.00	0.00	0.00	0.85	0.85	0.84	0.88	0.86	0.93	0.76	-	-	-	0.85	0.65
**8**	-22244	-22545	0.06	0.14	0.14	0.33	0.19	0.08	0.07	0.00	0.00	0.00	0.83	0.81	0.87	0.84	0.81	0.89	0.95	0.80	-	-	0.85	0.60
**9**	-22434	-22665	0.01	0.02	0.14	0.18	0.35	0.07	0.15	0.10	0.00	0.00	0.72	0.74	0.83	0.82	0.83	0.87	0.85	0.93	1.00	-	0.84	0.60
**10**	-22291	-22548	0.05	0.02	0.00	0.14	0.19	0.32	0.13	0.07	0.08	0.00	0.83	0.72	0.90	0.87	0.81	0.82	0.81	0.94	0.88	1.00	0.86	0.57
**Cubic**																								
**1**	-25178	-25230	1.00	0.00	0.00	0.00	0.00	0.00	0.00	0.00	0.00	0.00	1.00	-	-	-	-	-	-	-	-	-	1.00	1
**2**	-23513	-23613	0.69	0.31	0.00	0.00	0.00	0.00	0.00	0.00	0.00	0.00	0.97	0.93	-	-	-	-	-	-	-	-	0.95	0.90
**3**	-23047	-23195	0.60	0.19	0.21	0.00	0.00	0.00	0.00	0.00	0.00	0.00	0.94	0.90	0.92	-	-	-	-	-	-	-	0.92	0.82
**4**	-22935	-22833	0.10	0.54	0.17	0.19	0.00	0.00	0.00	0.00	0.00	0.00	0.83	0.91	0.91	0.92	-	-	-	-	-	-	0.89	0.73
**5**	-22481	-22635	0.14	0.28	0.39	0.09	0.09	0.00	0.00	0.00	0.00	0.00	0.87	0.87	0.89	0.89	0.94	-	-	-	-	-	0.89	0.68
**6**	-22339	-22474	0.14	0.20	0.10	0.32	0.08	0.16	0.00	0.00	0.00	0.00	0.83	0.81	0.86	0.86	0.95	0.87	-	-	-	-	0.86	0.66
**7**	-22282	-22512	0.01	0.16	0.39	0.12	0.12	0.13	0.08	0.00	0.00	0.00	0.83	0.84	0.89	0.83	0.84	0.86	0.92	-	-	-	0.86	0.66
**8**	-22151	-22574	0.11	0.00	0.30	0.09	0.22	0.08	0.05	0.13	0.00	0.00	0.85	0.85	0.85	0.85	0.81	0.89	0.94	0.83	-	-	0.86	0.63
**9**	-22156	-22323	0.05	0.00	0.14	0.31	0.20	0.11	0.07	0.04	0.07	0.00	0.85	0.90	0.81	0.85	0.81	0.86	0.92	0.95	0.83	-	0.86	0.60
**10**	-22067	-22400	0.05	0.02	0.05	0.00	0.13	0.31	0.07	0.18	0.11	0.04	0.73	0.85	0.95	0.81	0.82	0.83	0.81	0.84	0.95	0.90	0.85	0.57

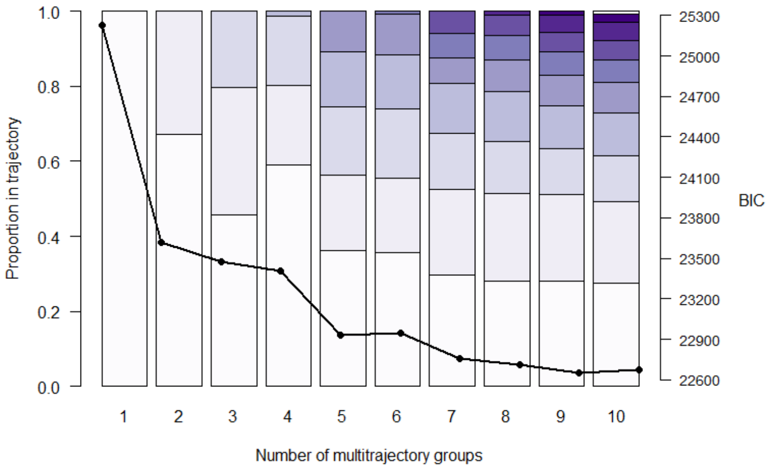

a)

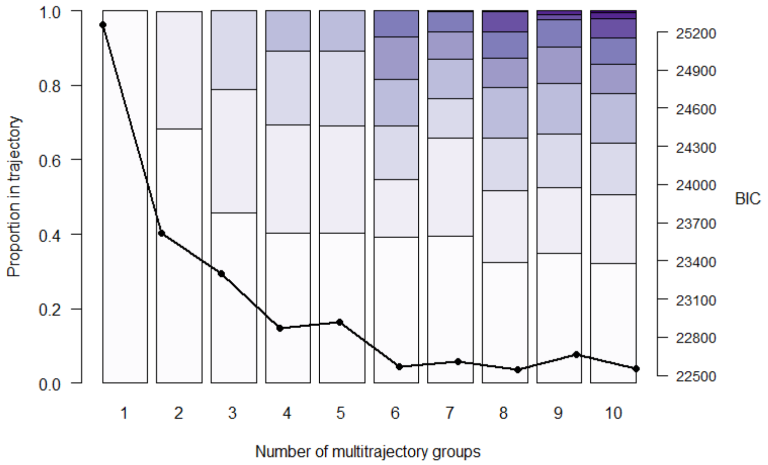

b)

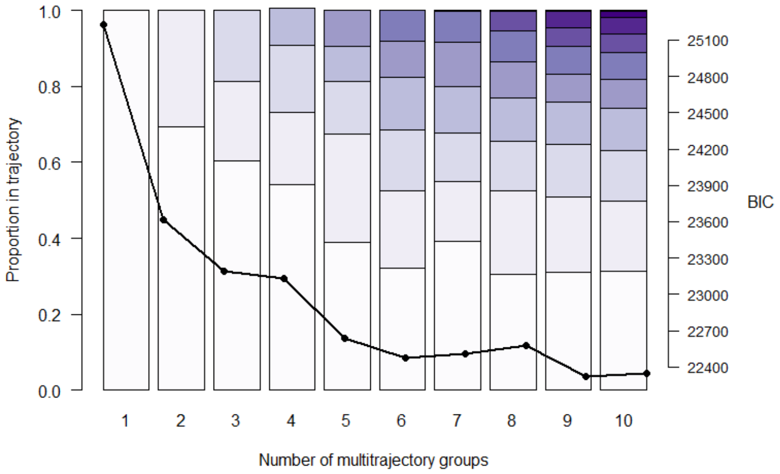

c)

Supplementary Figure 1. Model fit statistics and proportion of young people in each group when the number of trajectory groups are increased with a) Linear, b) Quadratic and c) Cubic polynomials

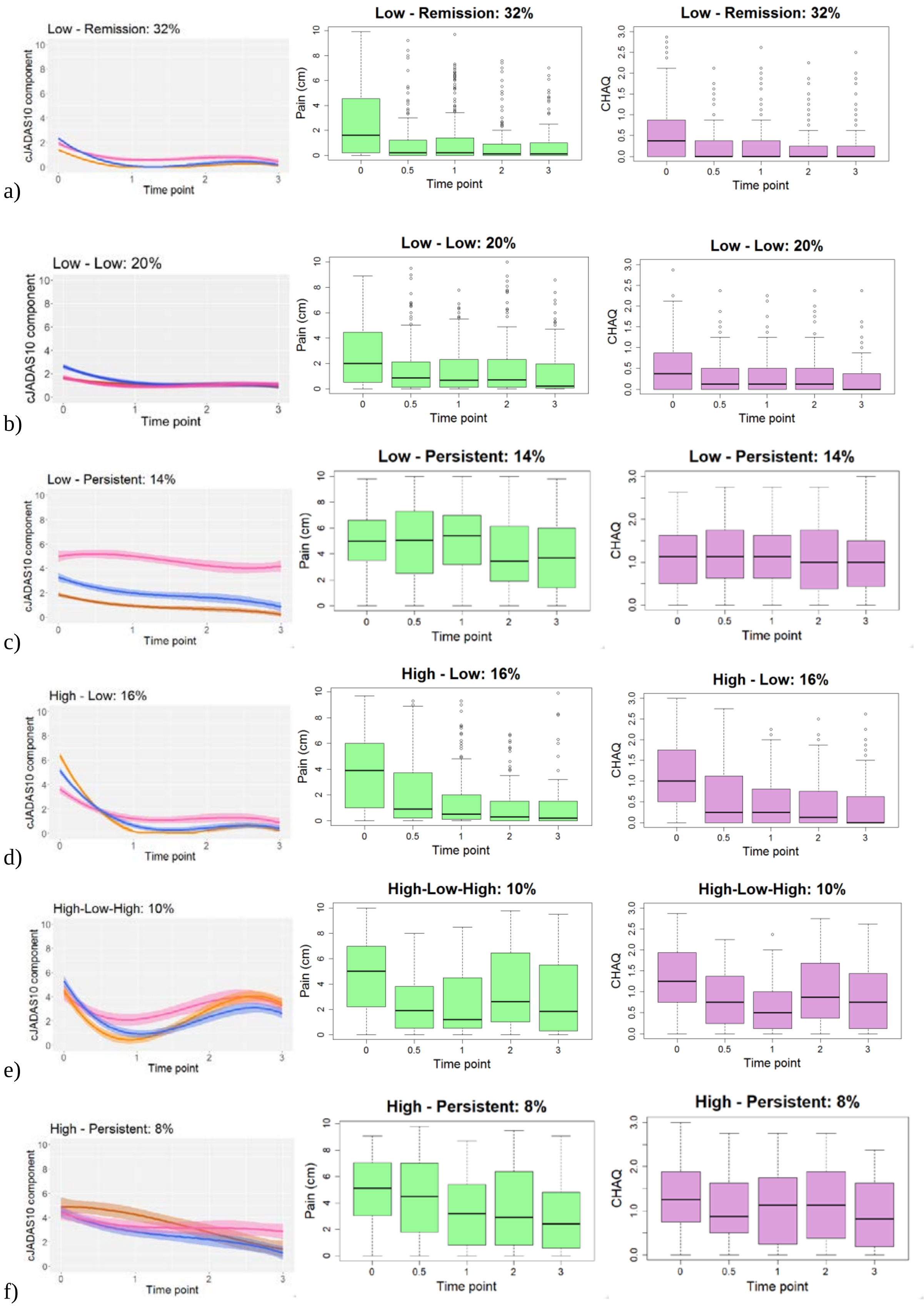

Supplementary Figure 2. Trajectories of cJADAS10 components compared with pain and CHAQ scores over the first three years following initial presentation to paediatric

rheumatology with JIA: a) Low-Remission, b) Low-Low, c) High-Low, d) High-Low-High, e) Low-Persistent, f) High-Persistent

Missing data analyses

CAPS collects informative drop-out data and it was likely that some outcome data were missing-not-at-random and may have been differentially distributed across trajectory groups. Cox proportional hazard models were constructed to test for differences in overall loss-to-follow-up and differences in young people discharged due to being 'well' across trajectory groups in the final multi-component outcome model. Where any differences were evident, single imputed values compared existing models with those constructed under 'most extreme scenario' analysis. Previous evidence has suggested that young people who fail to present to clinical appointments and those who are transferred to adult centres are more likely to be in remission, at least at the point of discharge [8, 35]. Therefore, for these models, young people who had been discharged due to i) being 'well', ii) repeat non-attendance and iii) transfer to adult care had cJADAS10 components imputed at zero at the time of discharge, as in previous work [8].

There was no difference in overall drop-out from the study between multivariate trajectory groups (Supplementary Table 3, Supplementary Figure 2). However, young people within the Low-Remission trajectory group were significantly more likely to be discharged 'well' compared with the other groups (Supplementary Table 3). Under the extreme scenario analysis, where those likely to be in remission had data imputed as such, there were no clinically significant differences in trajectory assignments or patterns compared to the original model. Therefore, since these models did not suggest bias from missing data in group allocation, no further imputation analyses were undertaken.

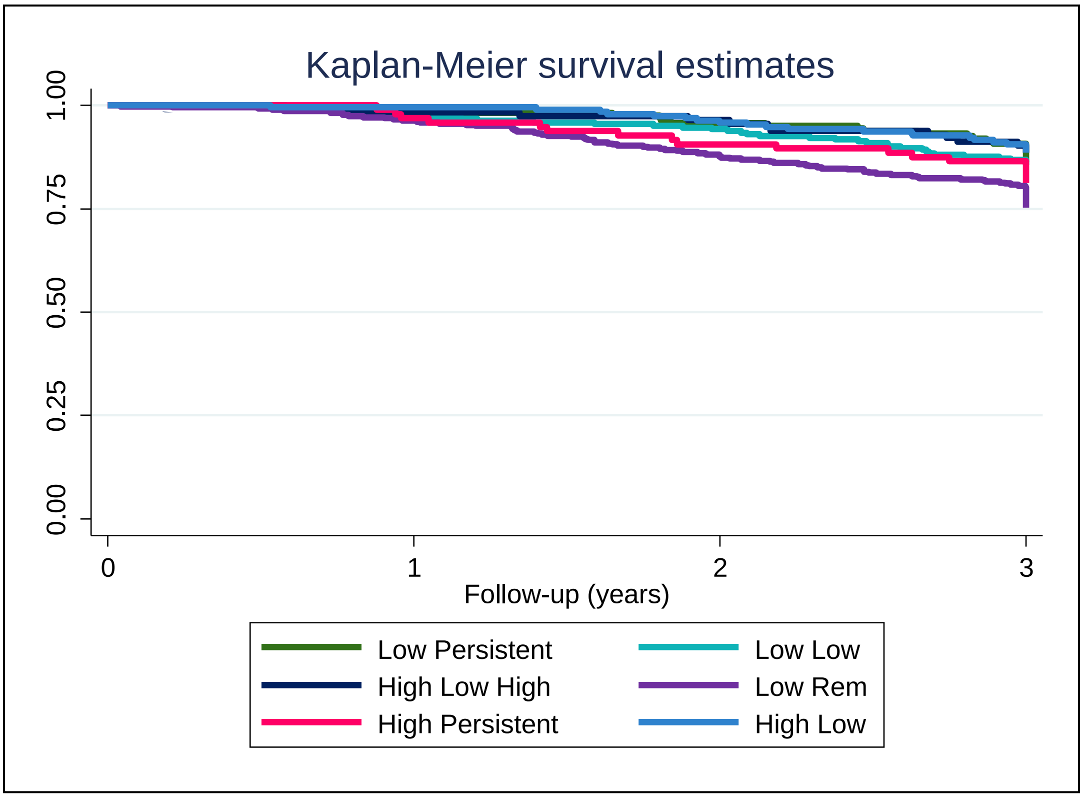

Supplementary Figure 3. Drop-out over the three years following initial presentation according to multi-trajectory group.

Supplementary Table 5. Differences in overall drop-out and discharge due to being 'well' across trajectory groups of overall cJADAS10 score

Trajectory group	Hazard Ratio (95% CI)	P-value
**Survival from overall drop-out**		
Low-Rem	Reference	Reference
Low-Low	1.2 (0.8, 1.7)	0.420
High-low	0.9 (0.6, 1.4)	0.579
High-low-High	0.9 (0.5, 1.7)	0.840
Low-Persistent	0.7 (0.5, 1.1)	0.170
High- Persistent	0.9 (0.5, 1.5)	0.623
**Survival from being discharged 'well'**		
Low-Rem	Reference	Reference
Low-Low	0.2 (0.1, 0.4)	<0.001

High-low	0.2 (0.1, 0.4)	<0.001
High-low-High	0.2 (0.8, 0.6)	0.003
Low- Persistent	0.2 (0.1, 0.4)	<0.001
High- Persistent	0.1 (<0.1, 0.5)	0.007

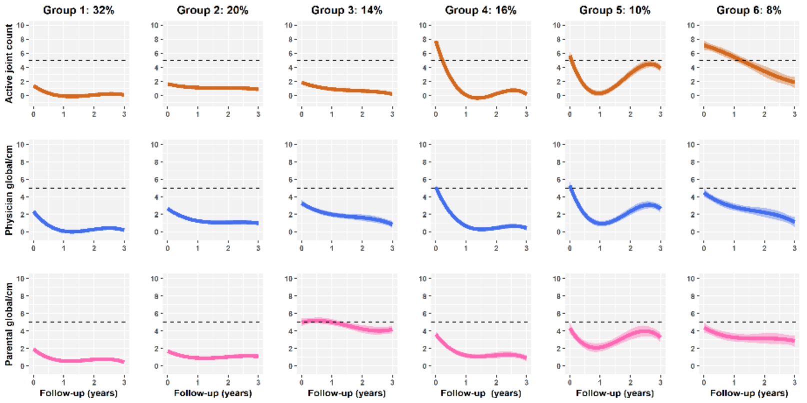
Group 1: 32%
Group 2: 20%
Group 3: 14%
Group 4: 16%
Group 5: 10%
Group 6: 8%
Active joint count
Physician global/cm
Parental global/cm
Follow-up (years)

a)

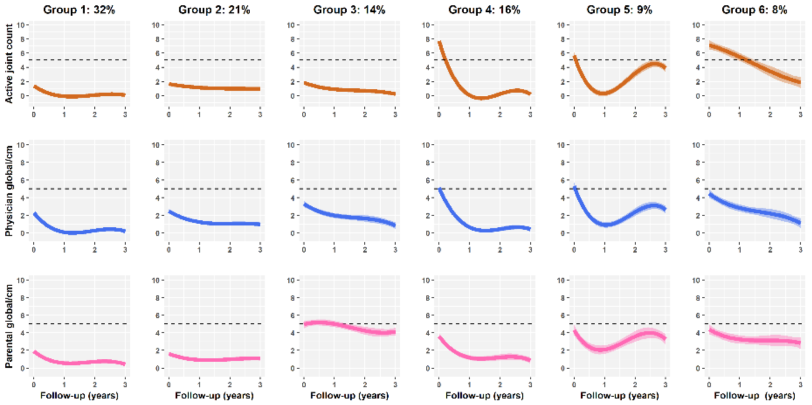
Group 1: 32%
Group 2: 21%
Group 3: 14%
Group 4: 16%
Group 5: 9%
Group 6: 8%
Active joint count
Physician global/cm
Parental global/cm
Follow-up (years)

b)

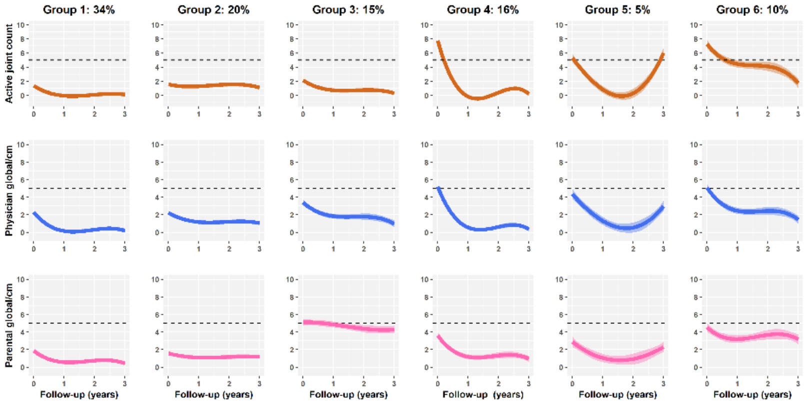
Group 1: 34%
Group 2: 20%
Group 3: 15%
Group 4: 16%
Group 5: 5%
Group 6: 10%
Active joint count
Physician global/cm
Parental global/cm
Follow-up (years)

c)

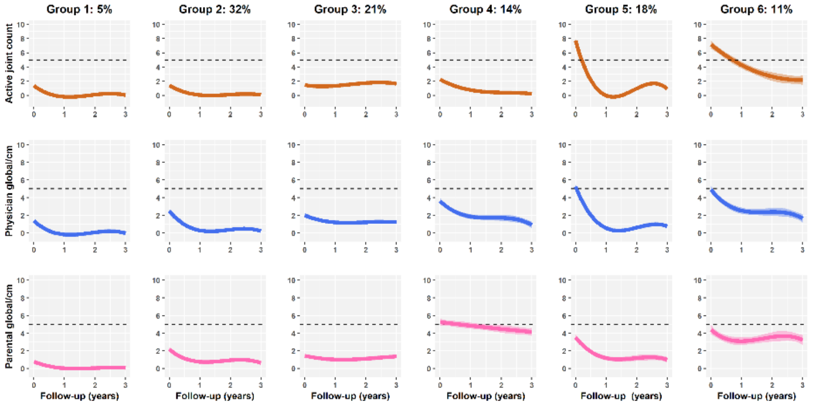
Group 1: 5%
Group 2: 32%
Group 3: 21%
Group 4: 14%
Group 5: 18%
Group 6: 11%
Active joint count
Physician global/cm
Parental global/cm
Follow-up (years)

d)

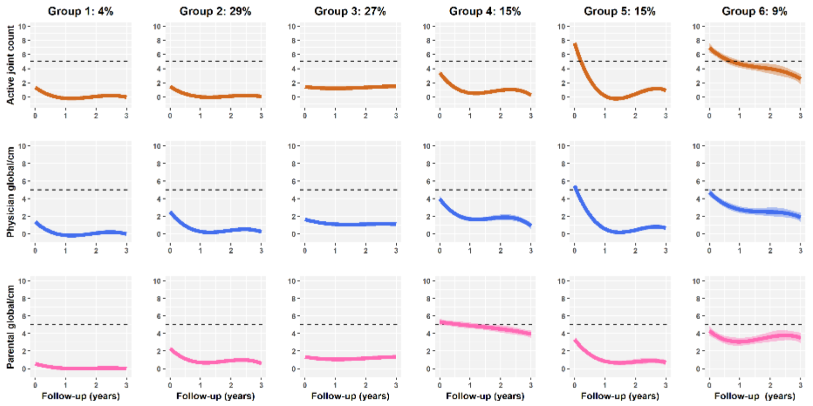

e)

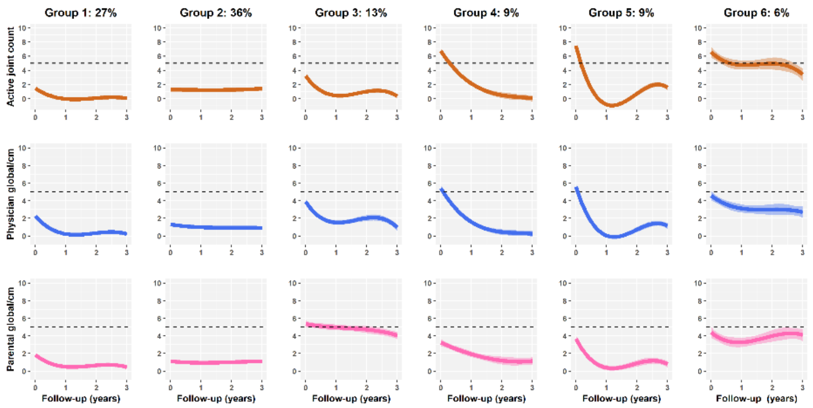

f)

Supplementary Figure 4. Multivariate trajectories of active joint counts, physician and parental global assessments with random noise added into the dataset a) 0% (original) b) 1%, c) 2.5%, d) 5%, e) 10%, f) 25%

Excerpt of code for group-based trajectory modelling

An example of code implemented in STATA 14 for the multivariate group-based trajectory modelling is given here. The following will build 6-class trajectory models with linear-only, quadratic-only and cubic-only polynomials within each loop. This was repeated, varying the number of classess from 1-10.

*Variable names

cjadas10_ajc= Active joint count limited from 0-10

physvas = Physician’s global assessment (cm)

parvas = Parental global evaluation of wellbeing (cm)

has_fup = takes the value of each follow-up

*Multivariate group-based trajectory models

```
Foreach i of numlist 1/3 {


traj, multgroups(6) ///

var(cjadas10_ajc0 cjadas10_ajc1 cjadas10_ajc2 cjadas10_ajc3 cjadas10_ajc4) ///

indep(has_fup0 has_fup1 has_fup2 has_fup3 has_fup4) ///

model(zip) ///

order(`i' `i' `i' `i' `i' `i') ///

min(0) max(10) ///

var2(physvas0 physvas1 physvas2 physvas3 physvas4) ///

indep2(has_fup0 has_fup1 has_fup2 has_fup3 has_fup4) ///

model2(cnorm) ///

order2(`i' `i' `i' `i' `i' `i') ///

min2(0) max2(10) ///

var3(parvas0 parvas1 parvas2 parvas3 parvas4) ///

indep3(has_fup0 has_fup1 has_fup2 has_fup3 has_fup4) ///

model3(cnorm) ///

order3(`i' `i' `i' `i' `i' `i') ///

min3(0) max3(10)


}
```

*Model evaluation

AIC and BIC (N=1184) were extracted (printed following trajetory code). Group sizes and average posterior probabilities of group membership were calculated using the using automatically produced _traj_Group and_traj_Prob variables:

```
tab _traj_Group

foreach i of numlist 1/6 {

sum _traj_ProbG`i’ if _traj_Group==`i’

}
```
